# Dynamics in kidney function markers as predictors of post-ICU survival: an innovative approach to enhance marker accuracy

**DOI:** 10.3389/fmed.2026.1765919

**Published:** 2026-06-22

**Authors:** Jakob Landau, Irene Unterman, Keren Tzukert, Maria Frantzi, Harald Mischak, Louis Boutin, François Depret, Agnieszka Latosinka, Yaron Ilan

**Affiliations:** 1 Department of Medicine, Hadassah Medical Center, Jerusalem, Israel; 2 Department of Nephrology, Hadassah Medical Center, Jerusalem, Israel; 3 Department of Dermatology and Venereology, Hadassah Medical Center, Jerusalem, Israel; 4 Mosaiques Diagnostics GmbH, Hannover, Germany; 5 INSERM, UMR-942, MASCOT, Cardiovascular Markers in Stress Condition, Université de Paris, Paris, France; 6 INSERM, UMR 1155, CORAKID, Tenon Hospital, Sorbonne Université, Paris, France; 7 Department of Anaesthesiology and Intensive Care, Hôpital Européen Georges Pompidou, AP-HP, 20 rue Leblanc, Paris, France; 8 Université Paris Cité, Paris, France; 9 FHU PROMICE AP-HP, Saint Louis and DMU Parabol, Critical Care Medicine and Burn Unit, AP-HP, Department of Anesthesiology, Université Paris Cité, Paris, France; 10 Inserm UMR-S 942, Paris, France; 11 INI-CRCT Network, Nancy, France

**Keywords:** AKI, biomarkers, dynamics, ICU, peptidome, variability

## Abstract

**Introduction and aims:**

The variability of physiological biomarkers is emerging as an independent indicator of health across various contexts. In intensive care unit (ICU) patients, high variability in serum creatinine, regardless of absolute values, has been associated with higher mortality. This study investigates the dynamics of blood and urine kidney function markers in ICU patients.

**Methods:**

In a *post hoc* analysis of the FROG-ICU cohort, focusing on patients with blood and urine samples at ICU admission and discharge, we investigate the relationships among kidney function markers, their dynamics between the two time points, and their association with kidney outcomes and survival. As a *post hoc*, exploratory analysis, all findings should be considered hypothesis-generating and require prospective validation.

**Results:**

At ICU admission, only serum Proenkephalin (PENK) values demonstrated a weak association with short-term post-ICU survival. In contrast, urine peptidome score CKD273 and cystatin C levels measured at ICU discharge were strongly associated with one-month survival. The differences in CKD273, Galectin-3, and plasma neutrophil gelatinase-associated lipocalin (NGAL) between admission and discharge were the most predictive of one-month survival, independent of baseline values. Furthermore, the slopes of CKD273 and plasma NGAL trajectories were prognostic. Absolute dynamic changes in urine NGAL were also linked to poorer short-term survival. Long-term post-ICU survival showed patterns similar to short-term outcomes with respect to biomarker differences during the ICU stay. When examining absolute changes, pronounced dynamics in PENK across the ICU course were associated with worse one-year survival.

**Conclusion:**

Both the magnitude and dynamics of kidney function markers may serve as independent predictors of short and long-term post-ICU mortality. As a *post hoc* exploratory study, this study is hypothesis-generating and requires prospective validation. It highlights the importance of tracking biomarker fluctuations in ICU patients rather than relying solely on static measurements.

## 1 Introduction

Patients admitted to Intensive Care Units (ICUs) face a higher risk of both early and long-term mortality (1). Diversity in etiologies leading to ICU admission, along with the varied interventions employed, hinders the accuracy of mortality predictions. Existing tools such as the Acute Physiology and Chronic Health Evaluation (APACHE) (2) and Sequential Organ Failure Assessment (SOFA) (3) Scores have been developed to assess the likelihood of in-ICU mortality. Yet, few tools are available for predicting long-term survival in survivors of critical illness. Identifying in-ICU subpopulations of patients at high risk of post-discharge mortality could enhance the quality of specific monitoring and facilitate the development of targeted post-discharge intervention strategies.

Acute kidney injury (AKI) is one of the most common complications in the ICU and is independently associated with higher mortality (4). Kidney function monitoring, therefore, can contribute to clinical decision-making and prognostic evaluation (5). According to current guidelines, serum creatinine levels and urine output are the primary biomarkers for monitoring kidney function. The Kidney Disease Improving Global Outcomes (KDIGO) guideline defines AKI (6). The severity of AKI is stratified according to KDIGO stage. However, creatinine levels are limited in reflecting kidney function during acute changes, due to their dependence on age, sex, and muscle mass, and a lag between the acute event and creatinine rise (7).

Numerous plasma and urine proteins have been investigated as potential biomarkers for kidney injury. Proenkephalin (PENK) is an endogenous opioid precursor protein that is freely filtered in the glomeruli and has been used as a predictor of AKI (8). Its stability and invariance to age and sex make it a reliable indicator of the Glomerular Filtration Rate (GFR) (9). Similarly, Cystatin C (CYSC) is freely filtered in glomeruli, reabsorbed, and catabolized by tubular cells; thus, its plasma levels reflect the glomerular filtration rate (10). In contrast, Neutrophil gelatinase-associated lipocalin (NGAL) is an acute-phase protein whose expression is increased during renal ischemia–reperfusion. Rather than a steady-state estimation of the glomerular filtration rate, it provides evidence of acute changes in kidney perfusion or kidney damage. Both serum and urine NGAL levels have been used to predict AKI (11). Galectin-3 is a lectin protein primarily expressed in kidney tubules and is involved in inflammation, with its expression upregulated in response to renal injury (12, 13).

As there are multiple causes and mechanisms of kidney dysfunction and damage in ICUs, multi-peptide risk stratification has garnered interest. Since most urinary proteins originate from the kidneys and urinary tract (14). Urine peptidomics-based classifiers are valuable tools for assessing kidney function. AKI204 and CKD273 are urinary peptidomics-based biomarker panels for detecting acute kidney injury and chronic kidney disease, respectively (15). They rely on the measurements of hundreds of naturally occurring peptides combined into a single score using a support vector machine (SVM). It enables them to detect kidney insults from diverse etiologies (15).

Given the interrelationship between AKI and ICU mortality, it is not surprising that many serum and urine markers of kidney function have dual roles as early prognostic markers for survival (Table 1). For example, AKI204 and CKD273 peptidome scores have recently been linked to a combined endpoint of death, kidney failure, or respiratory insufficiency in small sample-size polytrauma patients (16).

**Table 1:** Plasma and urine markers of kidney function and their use as prognostic markers for survival.

Sample type	Marker	Kidney impairment	Mortality
Plasma	PENK	Glomerular filtration rate estimation (35)	Mortality in patients with sepsis (36)
	CYSC	Glomerular filtration rate estimation (37)	All-cause and cardiovascular mortality in the elderly population; Post-ICU survival (38)
	NGAL	Tubular damage (39)	Associated with mortality in ICU patients (31)
	Galectin-3	Acute kidney injury (13)	One-month mortality in ICU patients (40)
Urine	NGAL	Acute kidney injury (11)	
	AKI204	Acute kidney injury (15)	Mortality in polytrauma patients (16), early (in-hospital) mortality (15)
	CKD273	Chronic kidney disease (41)	Mortality in individuals with type 2 Diabetes Mellitus and microalbuminuria (42)

In ICUs, patients with variable serum creatinine levels have been shown to have higher mortality rates compared to patients with stable levels. It is also true for patients with a decline in serum creatinine, indicating that the magnitude of the change is clinically significant regardless of its direction (17).

The dynamics of kidney function markers and their prognostic implications have not been extensively studied. To date, no specific post-discharge interventions have been shown to benefit ICU survivors. Therefore, identifying different subpopulations may help identify patients likely to benefit from interventions after ICU discharge. Here, we aim to investigate whether changes in kidney function markers can inform mortality risk in patients who have survived critical illness. Specifically, we compared the dynamics of urine peptidomics scores with those of kidney function markers, including serum creatinine and individual serum and urine peptide markers.

## 2 Methods

### 2.1 Patients

The cohort included in this study has been previously described (1, 18). The French and European Outcome Registry in Intensive Care Units (FROG-ICU) is a prospective cohort study. The study included 2087 patients, of whom 1,570 were discharged alive from the ICU and followed for 1 year. The study was conducted in France and Belgium under Good Clinical Practice, Declaration of Helsinki 2002, validated by the ethical committee (Comité de Protection des Personnes - Ile de France IV, IRB no. 00003835. Comission d’éthique biomédicale hospitalo-facultaire de l’hôpital de Louvain, IRB no. B403201213352) and was registered on ClinicalTrials.gov (NCT01367093) (19).

Each patient had blood and urine samples collected at ICU admission, and additional samples were collected at ICU discharge. Here, the inclusion criteria were that at least one additional blood and urine sample was collected at ICU discharge (*n* = 886). Exclusion criteria were: (i) Patients without a recorded date of last visit (*n* = 5). (ii) Patients with the second sample were taken on the same day as the first (*n* = 2). After applying these criteria, 879 patients were included in the final analysis.

Kidney function markers in blood and urine were collected at inclusion (T1) and ICU discharge (T2). Urine proteomics was performed as previously described (15). AKI status during the first 7 days of ICU hospitalization and KDIGO stage were defined as previously described, based on serum creatinine levels and urine output (20). Twenty-four patients had a second sample taken in anticipation of discharge, but subsequently deteriorated and died in the ICU before discharge occurred. Crucially, T2 sampling in the FROG-ICU study was performed based on the clinical judgment that these patients were approaching discharge, and the subsequent deterioration was unforeseen at the time of sampling. The vast majority of patients who died during the ICU course did not have a T2 sample collected at all; these twenty-four patients represent a clinically distinct group in whom imminent discharge was expected, and their early post-T2 deaths are precisely the high-risk outcomes this study aims to predict. They were therefore retained in the primary analysis. To assess the influence of this decision, all primary Cox analyses were repeated in the cohort restricted to the 855 patients who survived to ICU discharge (Supplementary Table S1). Most findings were consistent with the primary analysis; however, in the restricted cohort, some findings were not significant after FDR correction, suggesting these associations are partly driven by the earliest and highest-risk cases.

### 2.2 Statistical analyses

Associations between continuous variables were examined using Pearson’s correlation coefficient. Multinomial logistic regression was used to investigate the association between kidney function markers, clinical variables, and the AKI KDIGO stage. As the KDIGO definition depends on creatinine levels, serum creatinine was excluded from this analysis. Due to collinearity between AKI204 and CKD273, separate analyses were conducted for each. CKD273 analyses demonstrated superior statistical performance and prognostic value; therefore, it was selected for inclusion in the final models presented in the paper. Systolic and diastolic blood pressure measurements were condensed into mean arterial pressure (MAP). Missing blood pressure values (only 4.2% of our cohort) were imputed using the mean. Body Mass Index (BMI) was excluded from the analysis due to a high proportion of missing data (32.4%) and the absence of a difference in 30-day survival between survivors and non-survivors (Table 2).

**Table 2:** Patient characteristics.

Variable	Survived (Mean ± SD)	Died (Mean ± SD)	*P*-value
Number of Patients	793 (90.22%)	86 (9.78%)	
Age (years)	57.80 ± 16.83	69.99 ± 13.64	0.0000
BMI (kg/m^2)	27.30 ± 6.61	27.74 ± 5.84	0.6378
Systolic blood pressure (mmHg)	124.81 ± 22.13	121.45 ± 20.19	0.1829
Diastolic blood pressure (mmHg)	63.56 ± 13.97	60.58 ± 12.19	0.0620
ICU days	13.16 ± 16.94	19.09 ± 26.44	0.0040
KDIGO stage (0,1,2,3)	0.68 ± 1.1	1.29 ± 13.64	0.0000
Creatinine at T1 (umol/L)	100.20 ± 75.36	133.04 ± 99.63	0.0002
Plasma Proenkephalin A at T1 (pmol/lL)	69.19 ± 64.18	117.42 ± 103.50	0.0000
Plasma Cystatin C at T1 (mg/L)	1.34 ± 0.82	1.90 ± 0.99	0.0000
Plasma NGAL at T1 (ng/ML)	279.94 ± 379.72	398.82 ± 333.31	0.0060
Urinary NGAL at T1 (ng/ML)	340.25 ± 484.58	472.35 ± 569.21	0.0186
Plasma Galectin3 at T1 (ng/ML)	21.70 ± 12.90	30.19 ± 17.63	0.0000
CKD273 at T1	0.39 ± 0.41	0.57 ± 0.30	0.0001
AKI204 at T1	−0.09 ± 0.75	0.20 ± 0.69	0.0008
Creatinine T2 (umol/L)	79.65 ± 58.51	109.19 ± 85.81	0.0000
Plasma Proenkephalin A T2 (pmol/lL)	75.33 ± 71.08	131.47 ± 133.63	0.0000
Plasma Cystatin C at T2 (mg/L)	1.33 ± 0.74	2.06 ± 0.97	0.0000
Plasma NGAL at T2 (ng/ML)	165.71 ± 218.43	307.05 ± 314.12	0.0000
Urinary NGAL at T2 (ng/ML)	147.77 ± 289.17	264.93 ± 368.36	0.0006
Plasma Galectin3 at T2 (ng/ML)	20.71 ± 11.03	32.71 ± 21.55	0.0000
CKD273 at T2	0.22 ± 0.42	0.54 ± 0.28	0.0000
AKI204 at T2	−0.42 ± 0.68	−0.02 ± 0.64	0.0000

Results are expressed as counts (percentages) or means (± standard deviations). BMI body mass index. The p-value is calculated using a two-tailed T-test for independent samples to compare 30-day survivors and non-survivors.

Cox Proportional Hazards models were used to estimate the effects of kidney function markers and clinical variables on mortality following ICU discharge. Baseline clinical features included age, gender, hypertension, diabetes, chronic heart failure, and chronic kidney disease. In addition, severity parameters were included in the model: mean arterial pressure on ICU admission, acute kidney injury severity defined by KDIGO stage, and ICU stay in days. Our cohort did not include baseline severity scores or information on treatment during ICU (including vasopressors, mechanical ventilation, and renal replacement therapy). For overall mortality, survival time was defined as the interval from the second sample (T2) to the date of the last visit, in days. Patients without a known death status were censored on the date of their previous visit. Due to collinearity between CYSC, AKI204, and CKD273, separate models were constructed for each marker. Our primary metric is 30-day survival, as estimated by the CKD273 marker model. For the one-month mortality analysis, the survival duration was defined as the interval between the second sample (T2) and the date of the last visit, in days or 30 days. The difference in urinary function markers was defined as the second measurement (T2) minus the first (T1), and this raw difference was normalized using Z-scores. The difference slope was calculated as the raw difference divided by the number of ICU days between measurements, and the resulting slope values were normalized using Z-scores. Absolute difference was defined as the absolute value of the raw difference, which was also normalized using Z-scores.

To assess multicollinearity among kidney biomarkers, we calculated Variance Inflation Factors (VIFs) using Python (statsmodels library). The analysis included both baseline values at ICU admission (T1) and the corresponding z-score differences between admission and discharge. Rows with missing values were excluded before analysis. A threshold of 5 was used to indicate moderate collinearity. The same VIF analysis was additionally performed for models that included ICU admission (T1), ICU discharge (T2), and the corresponding z-scored absolute difference terms, simultaneously, to assess collinearity in the T2 + absolute difference sensitivity models. The area under the receiver operating characteristic (ROC) curve (AUC) was calculated in Python using the scikit-learn library, based on observed outcomes and model-predicted probabilities.

Biomarker differences between ICU admission (T1) and discharge (T2) were z-score normalized and stratified into quartiles using pandas.Qcut. For each quartile, 30-day mortality proportions and mean ±95% CI values at T1 and T2 were calculated. Pairwise comparisons of mortality between quartiles were performed using the two-sided Mann–Whitney U test using scipy.stats. Quartile distributions were visualized with normalized density histograms (seaborn.histplot), color-coded by quartile.

All statistical analyses were performed in Python 3.7.3, including *scipy* 1.7.3 for correlations, statistical tests, and clustering; sklearn 1.0.2 for regression analysis; lifelines 0.27.8 for survival analysis; and *Matplotlib 3.5.3* and *Seaborn 0.12.2* for plotting.

## 3 Results

### 3.1 Study population

Eight hundred seventy-nine patients met the inclusion and exclusion criteria as detailed in the Methods. Compared to the entire FROG-ICU cohort, the included patients were slightly younger (median age 60 years vs. 61 years in the entire cohort), 65.6% were male (63.7% in the whole cohort), and the median BMI was 26.6 kg/m^2^ (26 kg/m^2^ in the entire cohort). Survival rates were similar to those in the whole cohort - approximately 10% died within 1 month, and 21% died within 1 year, for a total of 184 deaths. Compared to survivors, 30-day non-survivors were older and had longer ICU stays (Table 2). BMI and blood pressure were not significantly altered. At both time points, all kidney function markers differed between 30-day survivors and non-survivors (Table 2).

### 3.2 Kidney function markers

**Figure 1:**
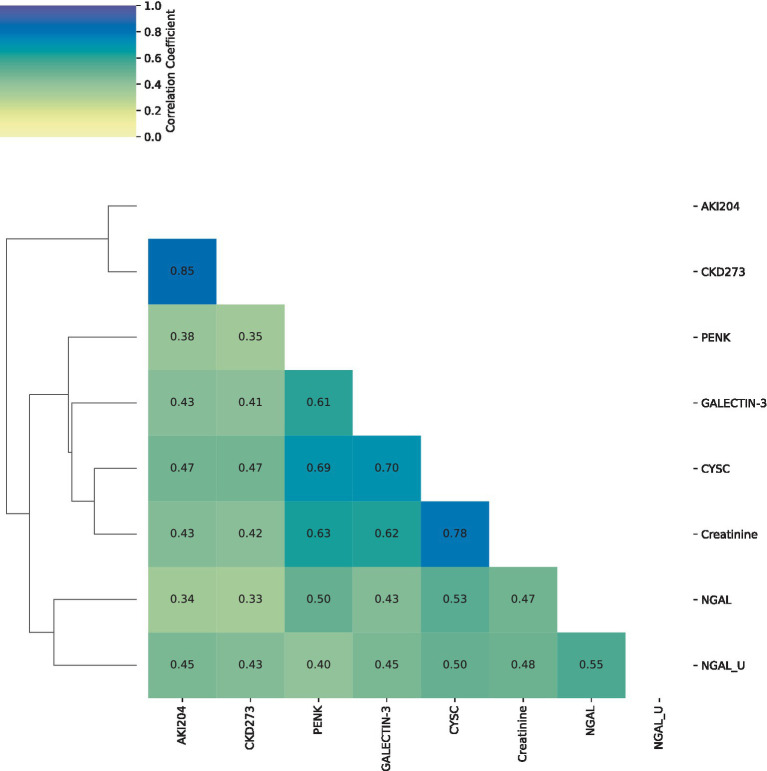
Pearson correlation between serum and urinary biomarkers at admission. The heatmap displays the pairwise correlation coefficients among eight biomarkers measured at ICU admission. Color intensity reflects the strength of correlation, ranging from low (yellow) to high (blue). Numerical values within each cell represent the Pearson *r* coefficient.

Kidney function markers in blood and urine were collected at ICU admission (T1) and ICU discharge (T2). At both time points, the blood marker proteins indicative of GFR clustered, including creatinine, PENK, and CYSC (Figure 1, Supplementary Figure 1). The acute-phase reactants NGAL and Galectin-3 did not cluster together, suggesting they may be involved in distinct mechanisms or pathways of kidney injury. The urinary peptidomics-based scores were highly correlated with one another, possibly due to overlap in the peptides they contain. The peptidome scores did not cluster with other markers. As expected, all correlations were positive.

To assess potential multicollinearity among the kidney biomarkers, we performed a Variance Inflation Factor (VIF) analysis (Supplementary Table S2). This evaluation demonstrated moderate collinearity between urinary peptidome scores (AKI204 and CKD273) and Cystatin C, indicating partial overlap in the information captured by these markers. In contrast, the dynamic measures (absolute differences between admission and follow-up values) showed little collinearity with their respective baseline levels. These results confirmed that although specific biomarkers share related variance, different measures provide independent prognostic information.

**Figure 2:**
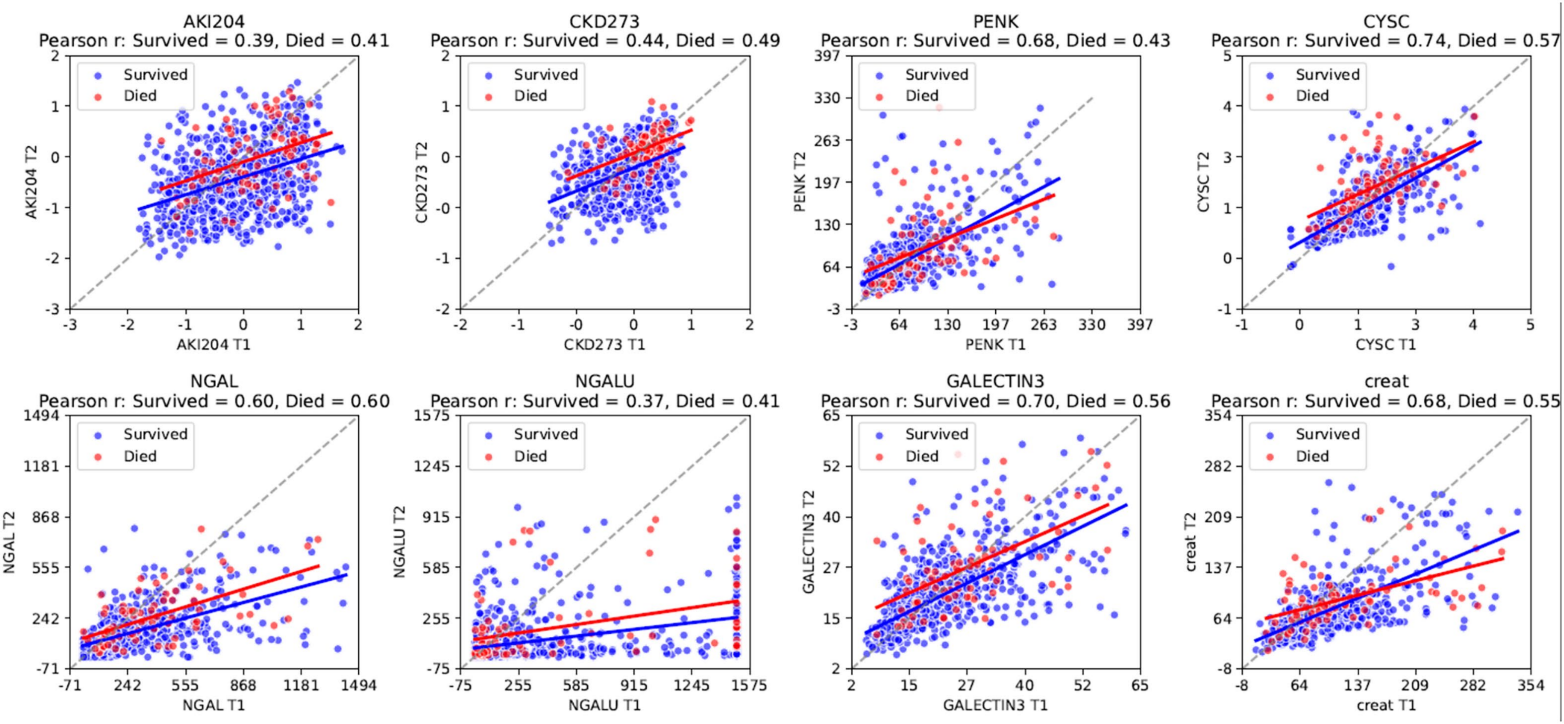
Pearson correlation of serum and urinary biomarkers between T1 and T2 in 30-day survivors and non-survivors. For each biomarker, scatter plots compare T1 and T2 values, stratified by survival status. Survivors are shown in blue and non-survivors in red. Regression lines were fitted separately for each group, and Pearson correlation coefficients (*r*) are reported to quantify the strength of association. A dashed line indicates a constant slope without variation between T1 and T2. Extreme outliers (>3 standard deviations from the mean) were excluded to improve interpretability.

While the markers clustered similarly at each time point, there was variability within each marker (Figure 2). The cluster of GFR indicators (CYSC, PENK, Creatinine) and Galectin-3 exhibited lower variance between time points. In contrast, the acute-phase reactants and multi-peptide scores exhibited greater variance, leading to a lower Pearson correlation between T1 and T2 values. There was a positive trend: kidney function improved between T1 and T2, and most markers of kidney damage decreased over time. Consequently, oversaturated NGAL values in urine observed at T1 were not observed at T2. A similar trend of improved kidney function was observed for non-survivors.

### 3.3 Kidney function markers and kidney outcomes

**Figure 3:**
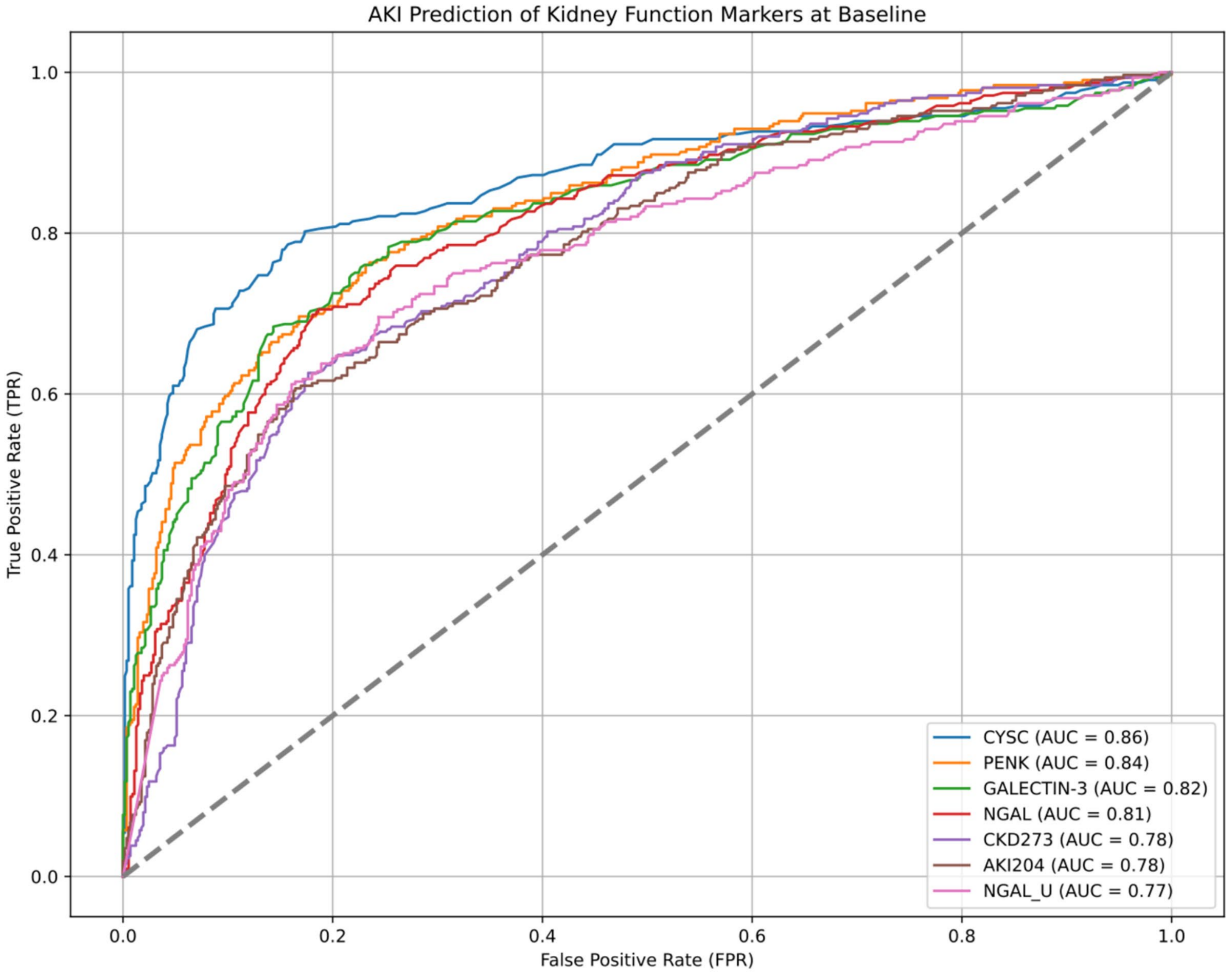
ROC-AUC of individual kidney function markers at admission for AKI prediction. Receiver operating characteristic (ROC) curves illustrate the diagnostic performance of each biomarker in predicting acute kidney injury (AKI) at baseline. The area under the curve (AUC) values quantify the predictive accuracy. The dashed diagonal line represents a random classifier with no predictive power.

Most kidney function markers performed similarly in predicting AKI according to KDIGO, with an area under the curve (AUC) of 0.77–0.86 (Figure 3). The most accurate marker was CYSC (Figure 3). CYSC, PENK, urine NGAL, and AKI204 were associated with mild and severe AKI (Supplementary Tables S3–S5). Additionally, Galectin-3 was associated with mild AKI (Supplementary Table S2). CKD273 was also associated with KDIGO AKI stage, to a lesser degree than AKI204, despite the score not being initially designed for this purpose (Supplementary Tables S6–S8).

### 3.4 ICU kidney function markers in the prediction of post-ICU mortality

**Figure 4:**
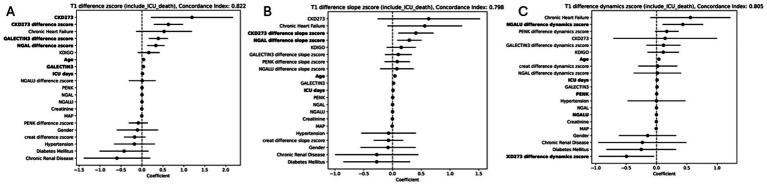
Cox regression coefficients for 30-day mortality including CKD273. Multivariable Cox proportional hazards models evaluating the association between kidney function markers and 30-day mortality. **(A)** Includes biomarker measurements at admission and the differences between T1 and T2. **(B)** Incorporates the slope of change over time. **(C)** Incorporates the absolute differences. Each forest plot displays hazard ratios with 95% confidence intervals. Variables with statistically significant associations (*p* < 0.05) are shown in bold. CKD273 was included in all models to assess its independent prognostic value.

We observed a bimodal pattern for survival, with 47% of deaths occurring within the first month after ICU discharge. Because the Variance Inflation Factor (VIF) analysis revealed moderate multicollinearity among kidney function biomarkers, particularly between urinary peptidome scores and Cystatin C, we performed separate Cox Proportional Hazards analyses for CKD273, AKI204, and Cystatin C to avoid instability of regression coefficients. To account for the increased risk of type I error due to multiple comparisons, we applied False Discovery Rate (FDR) correction to all biomarker-specific Cox models. Among the three biomarkers, CKD273 demonstrated the strongest prognostic performance for one-month mortality and is therefore presented in the main text (Figure 4). The corresponding analyses for AKI204 and Cystatin C are provided in the Supplementary Figures 2, 3. As a prespecified sensitivity analysis, all Cox models were rerun after excluding the 24 patients who provided a T2 sample in anticipation of discharge but died in the ICU before discharge (Supplementary Table S1). The majority of findings remained statistically significant in the restricted cohort. Exceptions were the CKD273 VALUES at T2, CKD273 difference term, serum NGAL slope of change, and the urine NGAL absolute difference, which did not reach FDR-adjusted significance after exclusion of these patients, indicating that the prognostic signal for these specific metrics is concentrated in, and partly dependent on, the highest-risk early-mortality cases.

At admission, the only biomarker associated with 30-day post-discharge survival was PENK, with a minimal coefficient and also non-significant after FDR adjustment in all three analyses (Supplementary Figure 4A, Supplementary Table 1). Of all discharge measurements, CKD273 showed the strongest association with 30-day survival, which remained significant after FDR adjustment (Supplementary Figure 4B, Supplementary Table 1). Adding the adjusted z-score difference to ICU discharge values did not add any prognostic value (Supplementary Figure 4C) (21).

Adding the ICU admission difference to the CKD273 model revealed that differences in CKD273, Galactin3, and plasma NGAL were most strongly associated with one-month survival, all of which were significant after FDR correction (Figure 4A, Supplementary Table 1). CKD273 was marginally significant in FDR, and Galactin3 was also significant in FDR (Supplementary Table 1). Same findings regarding difference in Galectin3, and plasma NGAL at AKI204 and Cystatin C analyses (Supplementary Figures 2A, 3A, Supplementary Table 1). and Cystatin C, Galactin3, and plasma NGAL at Cystatin. The association was also observed for CKD273 and plasma NGAL slope of change, i.e., the difference between T1 and T2 measurements normalized to the time interval between T1 and T2 (Figure 4B). After FDR correction, CKD273 slope was marginally significant, and NGAL slope was significant (Supplementary Table 1). The slope of change contains information about both the direction and rate of change, regardless of ICU stay length and the total difference. Urine NGAL absolute differences, reflecting marker dynamics during ICU, were associated with poor survival (Figure 4C). However, after FDR correction, they were only marginally significant (Supplementary Table 1). CKD273 absolute differences showed an inverse association; higher absolute differences were associated with better survival.

**Figure 5:**
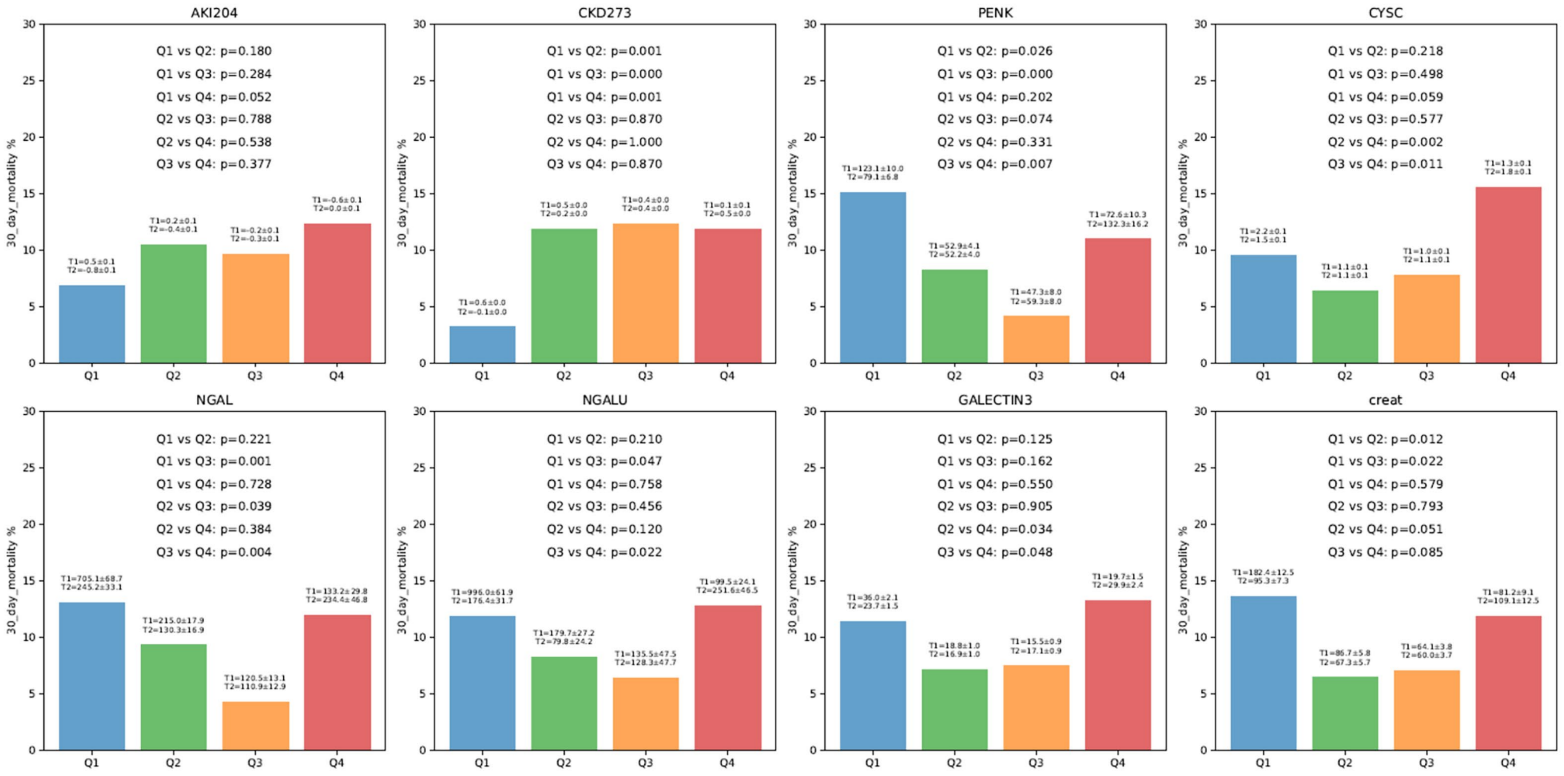
30-day mortality for biomarker difference *z*-scores across quartiles. For each biomarker, patients were divided into quartiles based on the *z*-score of the change between T1 and T2. Bar heights represent the percentage of patients who died within 30 days post-ICU discharge in each quartile. Pairwise comparisons between quartiles were assessed using the non-parametric Mann–Whitney *U* test, with *p*-values displayed above the corresponding bars. Mean values and confidence intervals for each biomarker at T1 and T2 were calculated within quartile groups. NGALU (urine NGAL). Creat (creatinine).

When stratified by quartiles, we observed a U-shaped association of creatinine, cystatin C, PENK, plasma NGAL, urine NGAL, and Galectin-3 with 30-day mortality (Figure 5). This pattern suggests that both improvement and deterioration in these markers were associated with increased mortality risk. It should be noted, however, that part of this U-shaped association may be explained by higher discharge (T2) values observed in quartiles 1 and 4 compared with quartiles 2 and 3, which could contribute to the apparent increased risk at both extremes. To disentangle the contribution of dynamic change from discharge-level values, we constructed Cox models for 30-day survival, including T2 value and absolute difference simultaneously (VIF < 5 for all terms, indicating acceptable collinearity) (Supplementary Figure 5). For most markers, including peptidome scores, PENK, cystatin C, plasma NGAL, and Galectin-3, the T2 value was the dominant predictor, suggesting that the U-shaped mortality pattern for these biomarkers is largely explained by elevated discharge levels at both extremes of change.

**Figure 6:**
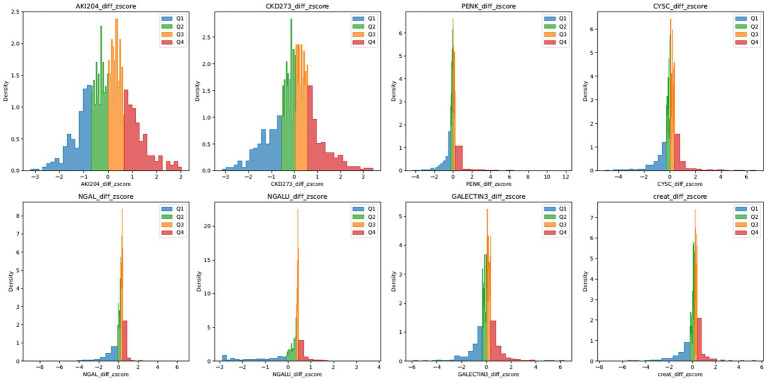
Distribution of biomarker difference *z*-scores across quartiles. For each biomarker, patients were divided into quartiles based on the *z*-score of the change between T1 and T2. Density plots show the distribution of these *z*-scores within each quartile, color-coded as follows: Q1 (blue), Q2 (green), Q3 (orange), and Q4 (red). This stratification enables visualization of how biomarker shifts vary across the population. NGALU (urine NGAL). Creat (creatinine).

**Figure 7:**
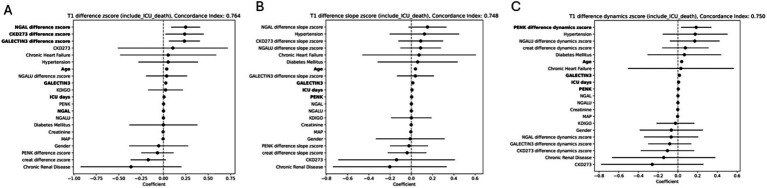
Cox regression coefficients for 365-day mortality including CKD273. Multivariable Cox proportional hazards models evaluating the association between kidney function markers and 365-day mortality. **(A)** Includes biomarker measurements at admission and the differences between T1 and T2. **(B)** Incorporates the slope of change over time. **(C)** Incorporates the absolute differences. Each forest plot displays hazard ratios with 95% confidence intervals. Variables with statistically significant associations (*p* < 0.05) are shown in bold. CKD273 was included in all models to assess its independent prognostic value.

In contrast, for urine NGAL, the absolute difference remained independently significant while the T2 value did not, indicating that the magnitude of dynamic change during the ICU course carries prognostic information beyond the discharge level itself. It confirms that the association between high urine NGAL dynamics and worse prognosis reflects true trajectory-dependent risk, rather than being an artifact of higher absolute values at discharge. In contrast, the polypeptide score AKI204 demonstrated a linear relationship (Figure 5): patients in quartile 1, reflecting score improvement (Figure 6, Supplementary Figure 6), had the most favorable outcomes, whereas those in quartile 4, reflecting score deterioration (Figure 6, Supplementary Figure 6), had the poorest outcomes. CKD273 exhibited a somewhat different pattern, suggestive of an inverse U-shaped association, with quartile 4 showing better survival than quartile 3, although this difference did not reach statistical significance. This observation is consistent with the Cox regression analysis, which showed that greater dynamics in CKD273 were significantly associated with improved outcomes (Figure 7).

We applied the same Cox Proportional Hazards modeling approach to evaluate 365-day mortality using CKD273 (Figure 7), AKI204 (Supplementary Figure 7), and Cystatin C (Supplementary Figure 8). In all analyses, admission Galectin-3, as well as differences in plasma NGAL and Galectin-3 during the ICU stay, were significantly associated with long-term mortality (Figure 6A, Supplementary Figures 7A, 8A). Specifically, Cystatin C admission levels and Cystatin C difference during the ICU course were significantly associated with long-term mortality; only the admission levels were significant after FDR correction (Supplementary Figure 7A). The CKD273 and AKI204 analyses did not provide additional information (Figure 6A, Supplementary Figure 8A). The slope did not carry significant prognostic meaning (Figure 6B, Supplementary Figures 7B, 8B). With respect to biomarker dynamics, the absolute difference in PENK was associated with poorer survival in all analyses, with marginal significance after FDR correction (Figure 6C, Supplementary Figures 7C, 8C). Additionally, it demonstrated a U-shaped relationship across quartiles of 365-day mortality (Supplementary Figure 9). For 365-day survival, the absolute difference was not a significant predictor when adjusted for the discharge marker’s value (Supplementary Figure 10).

## 4 Discussion

Our analysis suggests that kidney function markers capture different aspects of renal impairment; nearly all markers investigated were associated with AKI status and KDIGO stage, yet they did not exhibit strong intercorrelation. The strongest correlations observed at both time points were between the two multi-peptide scores, AKI204 and CKD273, likely due to peptide overlap, and between serum creatinine and CYSC levels. The good performance of cystatin C in AKI prediction is expected, as it is highly correlated with serum creatinine, which underlies the AKI definition (10). Most kidney function markers showed moderate correlations between the first and second time points. This finding highlights the importance of repeated marker testing in ICU patients, as it provides additional information.

Fluctuations in kidney function are common during ICU stays and may signal poor outcomes. Correspondingly, markers of kidney function can change dynamically between measurements. The direction and magnitude of change between two time-point measurements capture aspects of kidney function not available from single-point assessment. The constrained disorder principle (CDP) defines systems by their inherent variability, which is dynamic and allows adaptation to both internal and external environmental changes (22). According to the CDP, this variability is limited by dynamic boundaries; either excessive or insufficient variability can be associated with disease states. CDP-based machine learning intelligence systems may facilitate the optimization of therapies by leveraging this inherent biological noise (22).

Furthermore, the CDP can be used to develop variability-based diagnostic tools that view system variability as a means of enhancing the accuracy of diagnostic biomarkers (21). The CDP has been proposed as a theoretical framework for biological systems operating within bounded variability; however, a two-time-point design cannot formally characterize intra-individual variability in the CDP sense. Our metrics are therefore more precisely described as measuring directional and magnitude changes between ICU admission and discharge (23–27). The variability of biomarkers as predictors of pathology has been described across various systems, making them appealing potential predictors (21, 28–30).

Previous studies have shown that cardiac biomarkers improve the clinically based prediction of post-ICU mortality risk (1). Here, we found that kidney biomarkers may serve a similar purpose (12, 31). In our study, CKD273, plasma NGAL, and Galectin-3, and particularly their dynamics during the ICU stay, were prognostic not only for 1-month survival but also for 365-day mortality. Importantly, these associations were evaluated in multivariable Cox models adjusting for established clinical risk factors, including age, sex, diabetes mellitus, chronic heart failure, and chronic kidney disease, as well as ICU-derived severity parameters available in the FROG-ICU dataset, namely mean arterial pressure at admission, AKI severity by KDIGO stage, and length of ICU stay. The dynamic biomarker metrics demonstrated independent prognostic value beyond these clinical and severity variables, suggesting they capture pathophysiological information not reflected by conventional clinical parameters alone. We acknowledge that comprehensive severity scores, such as SOFA and APACHE, were not available in our dataset; whether dynamic kidney biomarkers provide incremental value beyond these scores warrants further evaluation in future studies. Specifically, a high rate of improvement in CKD273 during the ICU stay was associated with a better prognosis and prolonged 30-day survival, independent of absolute marker values. Conversely, a positive difference in CKD273 scores between ICU discharge and admission, reflecting deterioration in kidney function, was associated with a worse prognosis, regardless of absolute CKD273 levels at admission. It is a notable finding, as CKD273 was initially developed to predict chronic kidney disease rather than to serve as a prognostic marker of survival. These results suggest that peptidome scores are highly dynamic and that changes in these biomarkers carry crucial prognostic information. While the ICU course appears to influence short-term survival directly, persistent biomarker dynamics may reflect underlying processes that continue to shape long-term outcomes.

Interestingly, although absolute PENK and urine NGAL levels were not independently predictive of poor survival, the absolute difference between discharge and admission values was significantly associated with 30-day mortality for urine NGAL, while PENK dynamics were associated with 365-day mortality. When stratified by quartiles, PENK and urine NGAL showed a U-shaped relationship with 30-day and 365-day post-ICU discharge mortality, suggesting that both markedly significant and low changes may reflect underlying risk. It was further confirmed by Cox models adjusted for T2 values, in which the absolute difference remained independently significant, whereas T2 did not, ruling out the possibility that the U-shaped pattern is driven solely by elevated discharge-level values at the extremes. This finding aligns with previous studies on serum creatinine variability (17, 32).

For 365-day post-ICU discharge mortality, as for short-term mortality, both plasma NGAL and galectin 3 differences were significantly associated with worse outcomes. As for absolute dynamics, only the PENK absolute difference was associated with poor outcomes. PENK also showed a U-shaped relationship with 365-day mortality when stratified by absolute change, suggesting that both pronounced increases and decreases in PENK levels may indicate underlying renal instability. This pattern suggests that biomarker variability, rather than static values alone, can signal impaired renal reserve and heightened vulnerability over time.

The divergent association patterns observed across biomarkers, U-shaped for most GFR-related markers, linear for AKI204, and inverse U-shaped for CKD273, reflect fundamental differences in the biological meaning, confounding determinants, and inherent signal variability of each marker class, and should be interpreted accordingly rather than through a single framework. GFR-estimation markers such as PENK, cystatin C, and creatinine are influenced not only by glomerular filtration but also by non-renal factors, including muscle mass, inflammation, and fluid balance, all of which fluctuate substantially during critical illness. Consequently, changes in these markers between admission and discharge represent a composite of true renal recovery, hemodynamic shifts, and systemic inflammatory resolution. As demonstrated in our sensitivity analyses, the U-shaped mortality pattern for these markers is largely explained by higher absolute discharge-level values at both extremes of dynamic change; however, a residual variability component cannot be excluded. Urine NGAL represents a mechanistically distinct signal: as a highly sensitive and early marker of tubular epithelial injury, its levels can rise steeply within hours of a renal insult and resolve rapidly upon recovery, producing intrinsically high within-patient variability (33, 34). In this context, the magnitude of dynamic change in urine NGAL may capture the biological signal of acute tubular injury and recovery more faithfully than the absolute discharge value alone, which explains why the absolute difference remained an independent prognostic predictor in Cox models adjusted for T2 values, whereas T2 itself did not. Urinary peptidome scores (CKD273, AKI204), in contrast, are composite indices derived from hundreds of naturally occurring peptides combined via support vector machine classification. This multi-peptide aggregation inherently dampens individual-peptide noise, resulting in scores with lower intra-patient variability and greater measurement stability between time points. As a result, the change, specifically the direction and rate of peptidome score evolution across the ICU course, carries the dominant prognostic signal, rather than the magnitude of fluctuation per se. For CKD273, improvement in the score over the ICU stay was independently associated with better survival, consistent with progressive reduction in the chronic kidney disease–associated peptide signature; for AKI204, worsening was linearly associated with poorer outcomes. Together, these observations highlight that the prognostic significance of biomarker dynamics is inseparable from the underlying biology of each marker: the sources of variability, the confounders shaping absolute values, and the timescale of the biological process each marker reflects must all be considered when interpreting dynamic biomarker–outcome associations in ICU patients.

This study has several limitations. First, the *post hoc*, exploratory design means that no hypotheses were pre-specified and all reported associations must be considered hypothesis-generating rather than confirmatory; causal inference cannot be drawn from these data. Further, despite FDR correction, the large number of biomarker-derived analyses relative to the number of mortality events increases the risk of overfitting and residual false-positive findings. Therefore, all associations should be interpreted cautiously, and all findings require independent prospective validation before clinical application. The only documented outcome was survival, which is impacted by multiple factors. No specific information was collected on long-term kidney function outcomes. Also, our dataset included only two time points per patient, which may limit the ability to accurately capture the full variability or dynamic trajectory of biomarker changes over time. Importantly, a slope derived from only two measurements is mathematically equivalent to a scaled difference and cannot capture true intra-individual variability or a dynamic trajectory; future studies with serial, multi-time-point sampling are required. SOFA and APACHE severity scores were not available in a uniform format for this subset of patients, precluding formal adjustment for ICU severity indices and limiting the ability to demonstrate incremental prognostic value beyond established severity scores. Unmeasured confounding by vasopressor support, renal replacement therapy, mechanical ventilation, fluid balance, and diuretic use cannot be excluded.

In conclusion, this exploratory, hypothesis-generating post hoc analysis suggests that dynamic changes in kidney function markers between ICU admission and discharge may be associated with post-ICU short- and long-term mortality. All findings are exploratory and require prospective validation. Future studies should collect serial biomarker samples at multiple time points and should incorporate post-discharge renal endpoints, including eGFR trajectories, major adverse events, and mortality. Such designs would allow robust characterization of biomarker dynamics and direct comparison with established severity scores such as SOFA and APACHE, thereby establishing the true incremental prognostic value of kidney biomarker dynamics in the ICU setting.

## Data Availability

The raw data supporting the conclusions of this article will be made available by the authors, without undue reservation.
