# Supplementary material for: Dynamics in kidney function markers as predictors of post-ICU survival: an innovative approach to enhance marker accuracy
Source: Front Med (Lausanne). 2026 Jun 22;13:1765919. doi: 10.3389/fmed.2026.1765919 (PMC13333596; doi:10.3389/fmed.2026.1765919)
Supplement: Supplementary file 1 [file Supplementary_file_1.pdf]

Supplementary Tables

Supplementary Table 1. FDR test for the cohort with ICU deaths and without ICU deaths. (See enclosed Excel file).

Variable	VIF
const	12.96398916
**AKI_204seqBHa_T1**	**6.763736874**
**CYSC_T1**	**6.234579638**
**CKD273_T1**	**5.554982028**
NGAL_U_T1	4.775579715
AKI204_diff_zscore	4.218909987
NGAL_T1	3.73547655
NGALU_diff_zscore	3.535564858
CKD273_diff_zscore	3.444926248
csabad_creat_umol_L	3.293608125
GALECTIN3_T1	3.262492024
NGAL_diff_zscore	3.223840932
CYSC_diff_zscore	3.134992845
PENK_T1	3.104545064
GALECTIN3_diff_zscore	2.446486857
KDIGO1	2.297885308
PENK_diff_zscore	2.27680427

Supplementary Table 2. Variance Inflation Factor (VIF) analysis for multivariable models at T1. This table presents the VIF values calculated for all covariates included in the multivariable regression models at T1 and their difference to T2.

KDIGO=1	coef	std err	z	P>|z|	[0.025	0.975]
const	-1.1881	0.192	-6.191	0	-1.564	-0.812
**CYSC**	0.9914	0.254	3.909	0	0.494	1.488
**GALECTIN -3**	0.5803	0.178	3.258	0.001	0.231	0.929
**AKI204**	0.4629	0.15	3.087	0.002	0.169	0.757
**PENK**	0.6575	0.248	2.648	0.008	0.171	1.144
Chronic heart failure	0.7509	0.489	1.536	0.125	-0.207	1.709
Female Gender	-0.3739	0.256	-1.463	0.143	-0.875	0.127
Diabetes Mellitus	-0.3569	0.338	-1.057	0.29	-1.019	0.305
NGAL	0.1083	0.15	0.721	0.471	-0.186	0.403
BMI	0.0727	0.118	0.614	0.539	-0.159	0.305
NGAL_U	0.0736	0.137	0.535	0.592	-0.196	0.343
Hypertensi on	0.1275	0.261	0.487	0.626	-0.385	0.64
Age	-0.0607	0.142	-0.427	0.67	-0.34	0.218
MAP	0.0469	0.12	0.389	0.697	-0.189	0.283
Chronic renal disease	-0.0964	0.579	-0.167	0.868	-1.231	1.038

Supp. Table 3: Multinomial regression for KDIGO stage 1, with AKI204.

KDIGO=2	coef	std err	z	P>|z|	[0.025	0.975]
const	-3.0169	0.349	-8.641	0	-3.701	-2.333
**CYSC**	1.6461	0.291	5.662	0	1.076	2.216
**PENK**	0.8599	0.272	3.162	0.002	0.327	1.393
**AKI204**	0.6891	0.235	2.937	0.003	0.229	1.149
Age	-0.3764	0.212	-1.778	0.075	-0.791	0.039
Chronic heart failure	1.0091	0.607	1.663	0.096	-0.18	2.198
GALECTIN 3	0.3003	0.227	1.321	0.187	-0.145	0.746
NGAL_U	0.2203	0.179	1.23	0.219	-0.131	0.571
Female Gender	0.372	0.357	1.041	0.298	-0.328	1.072
Chronic renal disease	0.5989	0.641	0.935	0.35	-0.657	1.855
Hypertension	0.3738	0.403	0.927	0.354	-0.417	1.165
Diabetes Mellitus	-0.2464	0.448	-0.549	0.583	-1.125	0.632
NGAL	0.0996	0.188	0.529	0.597	-0.269	0.469
BMI	0.0802	0.161	0.499	0.618	-0.235	0.395
MAP	-0.0185	0.183	-0.101	0.919	-0.378	0.341

Supp. Table 4: Multinomial regression for KDIGO stage 2, with AKI204.

KDIGO=3	coef	std err	z	P>|z|	[0.025	0.975]
const	-1.5597	0.223	-7.003	0	-1.996	-1.123
**CYSC**	1.6198	0.261	6.217	0	1.109	2.13
**PENK**	1.0143	0.25	4.057	0	0.524	1.504
**NGAL U**	0.4789	0.139	3.449	0.001	0.207	0.751
**AKI204**	0.4106	0.175	2.349	0.019	0.068	0.753
**Age**	-0.3321	0.157	-2.109	0.035	-0.641	-0.024
Chronic heart failure	0.9161	0.531	1.724	0.085	-0.126	1.958
GALECTIN3	0.3264	0.191	1.713	0.087	-0.047	0.7
Chronic renal disease	0.9125	0.543	1.68	0.093	-0.152	1.977
BMI	0.1841	0.121	1.522	0.128	-0.053	0.421
Female Gender	-0.4047	0.292	-1.388	0.165	-0.976	0.167
MAP	-0.185	0.142	-1.301	0.193	-0.464	0.094
Hypertension	-0.3221	0.306	-1.052	0.293	-0.922	0.278
NGAL	0.1039	0.158	0.657	0.511	-0.206	0.414
Diabetes Mellitus	-0.2086	0.371	-0.563	0.574	-0.935	0.518

Supp table 5: Multinomial regression for KDIGO stage 3, with AKI204.

KDIGO=1	coef	std err	z	P>|z|	[0.025	0.975]
const	-1.217	0.193	-6.307	0	-1.595	-0.839
**CYSC**	0.9647	0.253	3.806	0	0.468	1.462
**GALECTIN3_**	0.5992	0.179	3.354	0.001	0.249	0.949
**PENK**	0.6787	0.247	2.751	0.006	0.195	1.162
**CKD273**	0.4085	0.172	2.373	0.018	0.071	0.746
Chronic heart failure	0.7223	0.484	1.494	0.135	-0.225	1.67
Female Gender	-0.3589	0.255	-1.408	0.159	-0.859	0.141
Diabetes Mellitus	-0.3451	0.335	-1.031	0.303	-1.001	0.311
NGAL	0.1215	0.149	0.815	0.415	-0.171	0.414
BMI	0.0851	0.117	0.728	0.467	-0.144	0.314
NGAL_U	0.0843	0.137	0.613	0.54	-0.185	0.354
Hypertension	0.156	0.26	0.6	0.548	-0.353	0.665
Chronic renal disease	-0.2422	0.566	-0.428	0.669	-1.352	0.867
Age	-0.0524	0.143	-0.366	0.714	-0.333	0.228
MAP	0.0331	0.12	0.275	0.783	-0.202	0.268

Supp. Table 6: Multinomial regression for KDIGO stage 1, with CKD273.

**KDIGO=2**	**coef**	**std err**	**z**	**P>|z|**	**[0.025**	**0.975]**
const	-3.0898	0.363	-8.518	0	-3.801	-2.379
**CYSC**	1.6035	0.291	5.507	0	1.033	2.174
**PENK**	0.8899	0.269	3.311	0.001	0.363	1.417
**CKD273**	0.7111	0.314	2.264	0.024	0.096	1.327
Age	-0.3683	0.213	-1.728	0.084	-0.786	0.05
Chronic heart failure	0.9778	0.602	1.623	0.105	-0.203	2.158
GALECTIN 3	0.3339	0.226	1.475	0.14	-0.11	0.778
NGAL_U	0.2361	0.178	1.324	0.186	-0.113	0.586
Female Gender	0.3866	0.357	1.083	0.279	-0.313	1.086
Hypertension	0.4144	0.402	1.031	0.303	-0.373	1.202
Chronic renal disease	0.4386	0.626	0.701	0.483	-0.788	1.665
NGAL	0.1137	0.187	0.609	0.542	-0.252	0.48
BMI	0.0865	0.16	0.54	0.589	-0.227	0.4
Diabetes Mellitus	-0.2368	0.447	-0.53	0.596	-1.113	0.639
MAP	-0.0437	0.182	-0.24	0.811	-0.401	0.314

Supp. Table 7: Multinomial regression for KDIGO stage 2, with CKD273.

KDIGO=3	coef	std err	z	P>|z|	[0.025	0.975]
const	-1.5547	0.222	-7.002	0	-1.99	-1.12
**CYSC**	1.6143	0.26	6.197	0	1.104	2.125
**PENK**	1.0396	0.247	4.205	0	0.555	1.524
**NGAL_U**	0.5034	0.139	3.611	0	0.23	0.777
**Age**	-0.316	0.158	-2.006	0.045	-0.625	-0.007
GALECTIN 3	0.3444	0.191	1.808	0.071	-0.029	0.718
Chronic heart failure	0.8898	0.526	1.69	0.091	-0.142	1.922
BMI	0.202	0.12	1.687	0.092	-0.033	0.437
MAP	-0.2044	0.141	-1.446	0.148	-0.481	0.073
Chronic renal disease	0.7542	0.529	1.426	0.154	-0.282	1.791
Female Gender	-0.3971	0.291	-1.364	0.173	-0.968	0.174
CKD273	0.2389	0.204	1.173	0.241	-0.16	0.638
Hypertension	-0.2921	0.305	-0.958	0.338	-0.89	0.306
NGAL	0.1164	0.157	0.74	0.46	-0.192	0.425
Diabetes Mellitus	-0.1905	0.368	-0.518	0.604	-0.911	0.53

Supp. Table 8: Multinomial regression for KDIGO stage 3, with CKD273.

Supplementary Figures

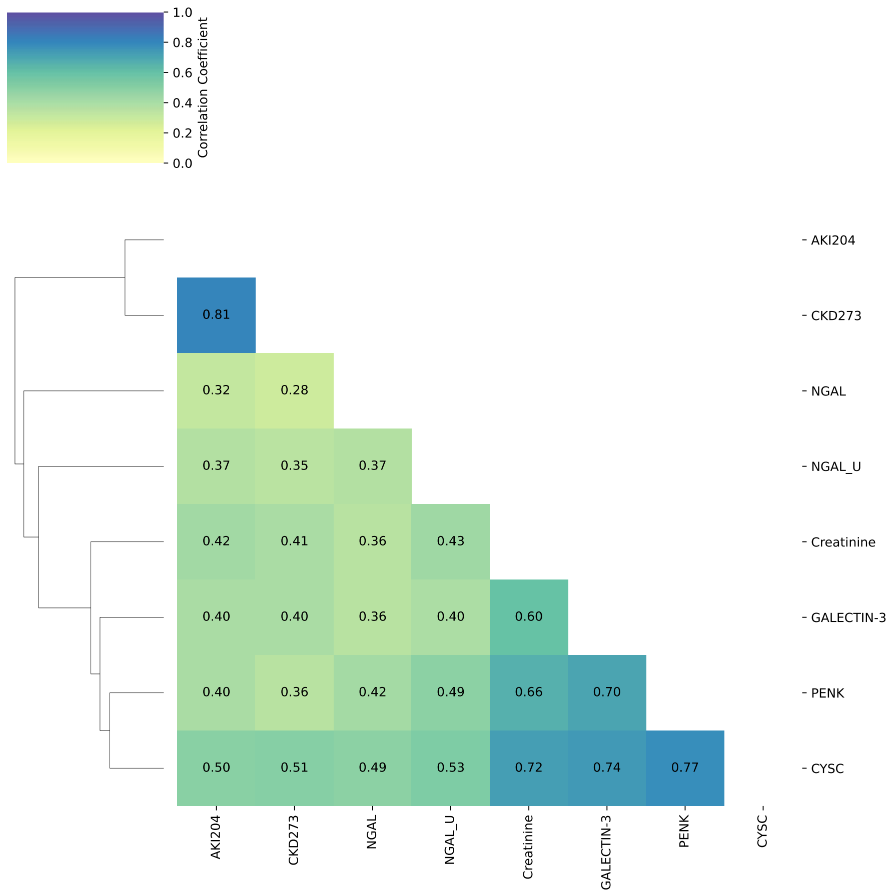

Supp. Figure 1: Pearson correlation between serum and urinary biomarkers at discharge. The heatmap displays the pairwise correlation coefficients among eight biomarkers measured at ICU discharge. Color intensity reflects the strength of correlation, ranging from low (yellow) to high (blue). Numerical values within each cell represent the Pearson r coefficient.

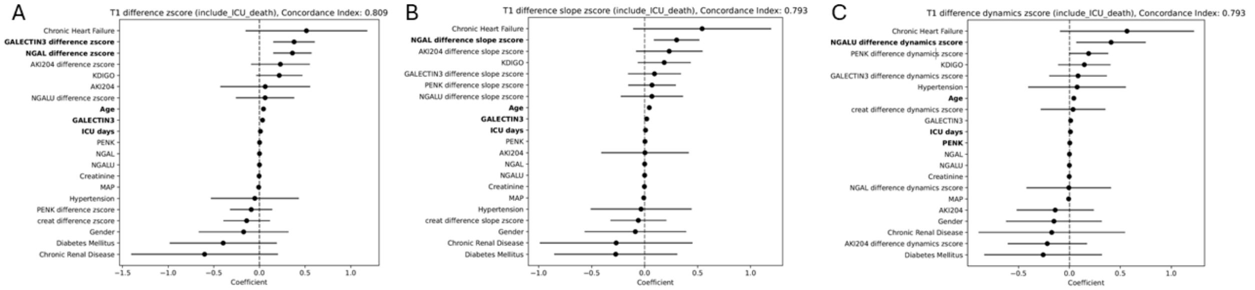

Supp. Figure 2: Cox regression coefficients for 30-day mortality including AKI204. Multivariable Cox proportional hazards models evaluating the association between kidney function markers and 30-day mortality. (A) includes biomarker measurements at admission and the differences between T1 and T2. (B) incorporates the slope of change over time. (C) incorporates the absolute differences. Each forest plot displays hazard ratios with 95% confidence intervals. Variables with statistically significant associations ($p < 0.05$) are shown in bold. AKI204 was included in all models to assess its independent prognostic value.

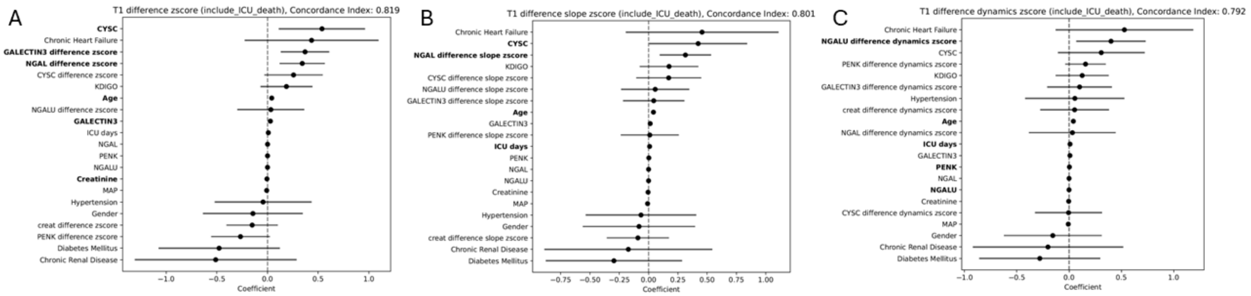

Supp. Figure 3: Cox regression coefficients for 30-day mortality, including Cystatin C. Multivariable Cox proportional hazards models evaluating the association between kidney function markers and 30-day mortality. (A) includes biomarker measurements at admission and the differences between T1 and T2. (B) incorporates the slope of change over time. (C) incorporates the absolute differences. Each forest plot displays hazard ratios with 95% confidence intervals. Variables with statistically significant associations ($p < 0.05$) are shown in bold. AKI204 was included in all models to assess its independent prognostic value.

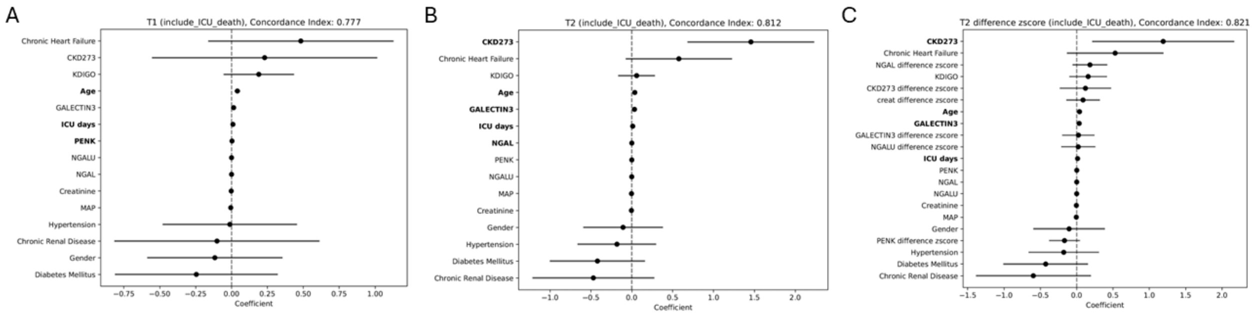

Supp. Figure 4: Additional Cox regression coefficients for 30-day mortality following ICU discharge, including CKD273. Forest plots display the results of three multivariable Cox regression models evaluating the association between kidney function markers and post-ICU mortality. (A) includes biomarker measurements at admission. (B) includes measurements at discharge. (C) incorporates discharge values along with changes during the ICU stay. Each plot shows hazard ratios with 95% confidence intervals. Variables with statistically significant associations ($p < 0.05$) are highlighted in bold. CKD273 was included in all models to assess its prognostic relevance across time points.

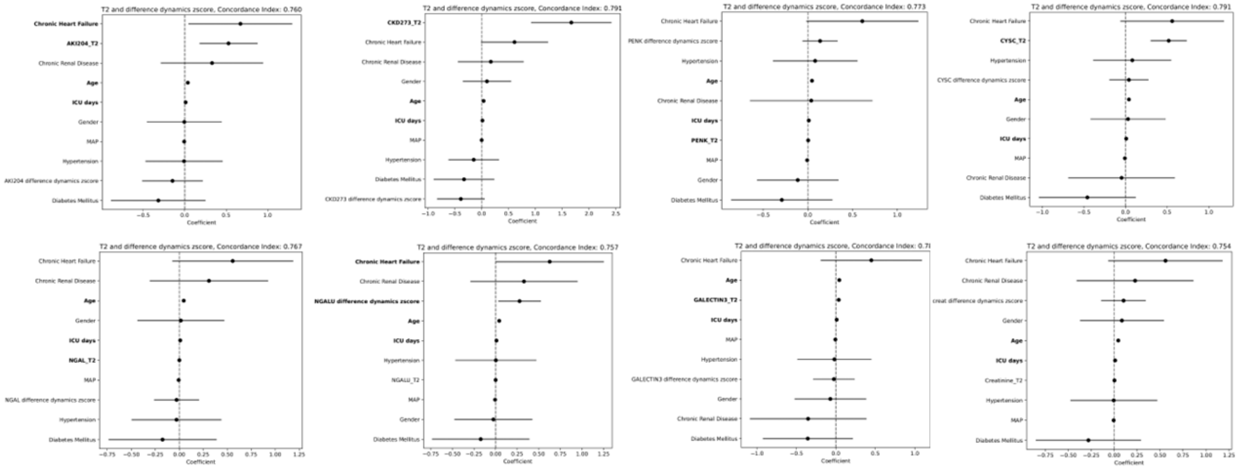

Supp. Figure 5: Cox regression coefficients for 30-day mortality, including T2 markers values and absolute T1 to T2 difference. Multivariable Cox proportional hazards models evaluating the association between kidney function markers and 30-day mortality. Each sub plot includes clinical features, different serum/urine markers, ICU discharge values, and absolute difference during ICU admission. Variables with statistically significant associations ($p < 0.05$) are shown in bold.

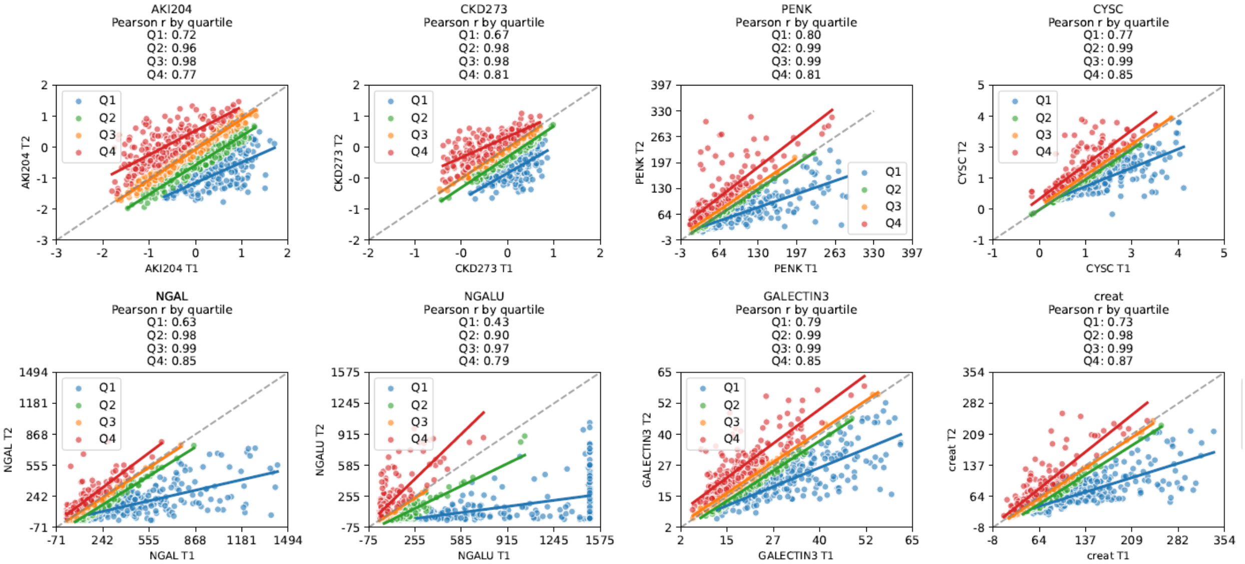

Supp. Figure 6: Correlation between T1 and T2 biomarker levels by markers difference z-scores across quartiles. For each biomarker, patients were divided into quartiles (Q1–Q4) based on the change between T1 and T2 z-scores. Scatter plots display the relationship between T1 and T2 values within each quartile, with regression lines fitted separately for each group. Pearson correlation coefficients (r) are reported per quartile to quantify the strength of association. A dashed line indicates a constant slope without variation between T1 and T2. NGALU (urine NGAL). Creat (creatinine).

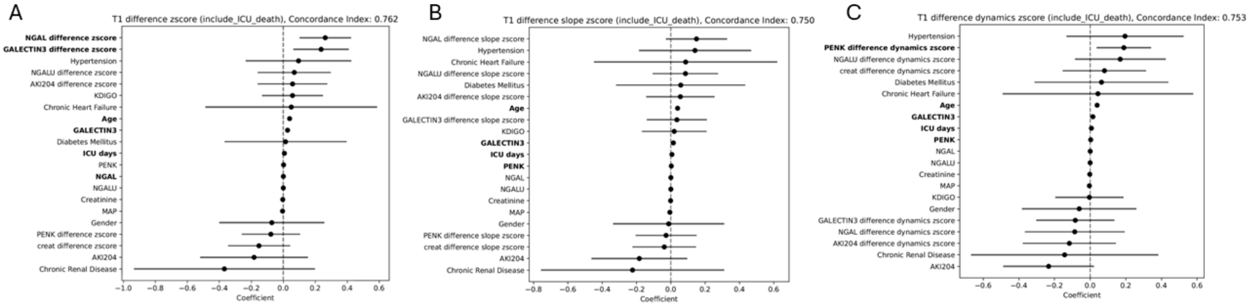

Supp. Figure 7: Cox regression coefficients for 365-day mortality including AKI204. Multivariable Cox proportional hazards models evaluating the association between kidney function markers and 365-day mortality. (A) includes biomarker measurements at admission and the differences between T1 and T2. (B) incorporates the slope of change over time. (C) incorporates the absolute differences. Each forest plot displays hazard ratios with 95% confidence intervals. Variables with statistically significant associations ($p < 0.05$) are shown in bold. AKI204 was included in all models to assess its independent prognostic value.

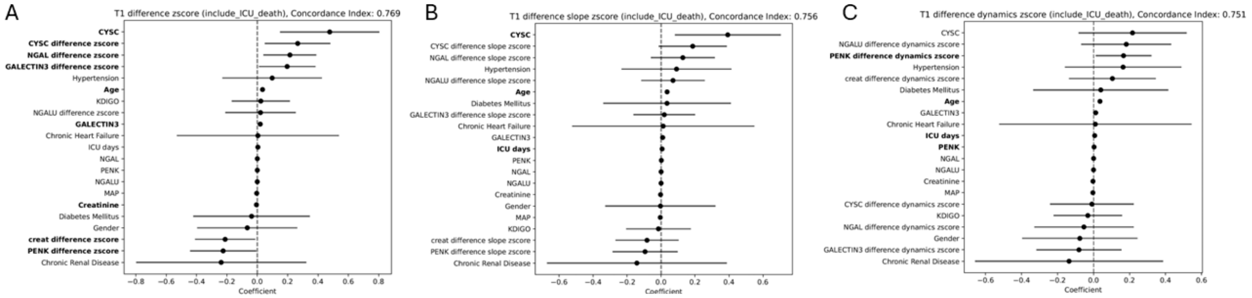

Supp. Figure 8: Cox regression coefficients for 365-day mortality, including Cystatin C. Multivariable Cox proportional hazards models evaluating the association between kidney function markers and 365-day mortality. (A) includes biomarker measurements at admission and the differences between T1 and T2. (B) incorporates the slope of change over time. (C) incorporates the absolute differences. Each forest plot displays hazard ratios with 95% confidence intervals. Variables with statistically significant associations ($p < 0.05$) are shown in bold. Cystatin C was included in all models to assess its independent prognostic value.

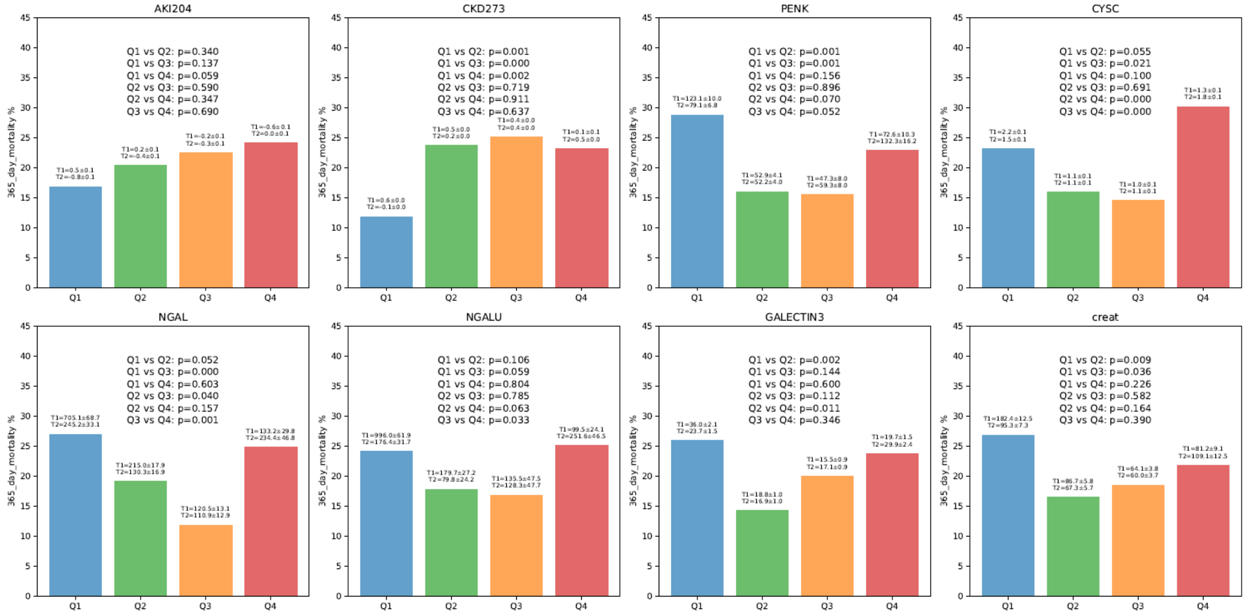

Supp. Figure 9: **365-day mortality for biomarker difference z-scores across quartiles.** For each biomarker, patients were divided into quartiles based on the z-score of the change between T1 and T2. Bar heights represent the percentage of patients who died within 30 days post-ICU discharge in each quartile. Pairwise comparisons between quartiles were assessed using the non-parametric Mann-Whitney U test, with p-values displayed above the corresponding bars. Mean values and confidence intervals for each biomarker at T1 and T2 were calculated within quartile groups. NGALU (urine NGAL). Creat (creatinine).

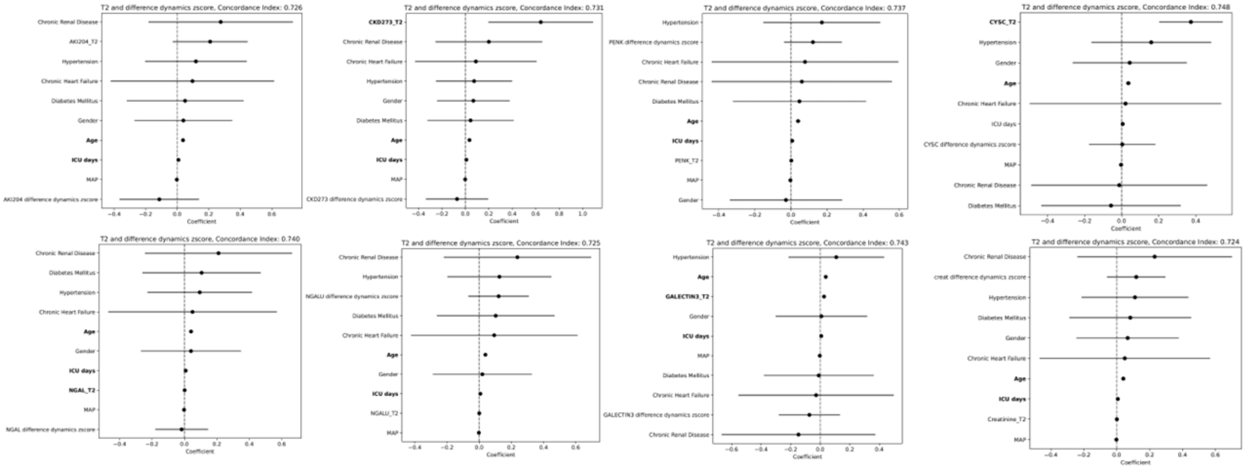

Supp. Figure 10: Cox regression coefficients for 365-day mortality, including T2 markers values and absolute T1 to T2 difference. Multivariable Cox proportional hazards models evaluating the association between kidney function markers and 30-day mortality. Each sub plot includes clinical features, different serum/urine marker's ICU discharge values and absolute differenc during ICU addmision. Variables with statistically significant associations ($p < 0.05$) are shown in bold.
